# GC/MS Analysis, Antioxidant Activity, and Antimicrobial Effect of *Pelargonium peltatum* (Geraniaceae)

**DOI:** 10.3390/molecules27113436

**Published:** 2022-05-26

**Authors:** Alan-Misael Alonso, Oscar Kevin Reyes-Maldonado, Ana María Puebla-Pérez, Martha Patricia Gallegos Arreola, Sandra Fabiola Velasco-Ramírez, Victor Zúñiga-Mayo, Rosa E. Sánchez-Fernández, Jorge-Iván Delgado-Saucedo, Gilberto Velázquez-Juárez

**Affiliations:** 1 Doctorado en Ciencias en Procesos Biotecnológicos, Centro Universitario de Ciencias Exactas e Ingenierías, Universidad de Guadalajara, Blvd. Marcelino García Barragán #1421, Guadalajara CP 44340, Jalisco, Mexico; alan.ealonso@alumnos.udg.mx; 2 Centro Universitario de Ciencias Exactas e Ingenierías, Laboratorio de Bioquímica Avanzada, Departamento de Química, Universidad de Guadalajara, Blvd. Marcelino García Barragán #1421, Guadalajara CP 44430, Jalisco, Mexico; oscar.reyes@alumnos.udg.mx (O.K.R.-M.); sandra.vramirez@academicos.udg.mx (S.F.V.-R.); 3 Centro Universitario de Ciencias Exactas e Ingenierías, Departamento de Farmacobiología, Universidad de Guadalajara, Blvd. Marcelino García Barragán #1421, Guadalajara CP 44430, Jalisco, Mexico; ampueblap@yahoo.com.mx; 4 Centro de Investigación Biomédica de Occidente, División de Genética, I.M.S.S., Sierra Mojada 800, Independencia Oriente, Guadalajara CP 44340, Jalisco, Mexico; marthapatriciagallegos08@gmail.com; 5 Campus Montecillo, CONACyT-Instituto de Fitosanidad, Colegio de Postgraduados, Texcoco CP 56230, Estado de Mexico, Mexico; zuniga.victor@colpos.mx; 6 Laboratorio Nacional de Investigación y Servicio Agroalimentario y Forestal (LANISAF), Universidad Autónoma Chapingo, Mexico-Texcoco km 38.5, Texcoco CP 56230, Mexico; resf2012@gmail.com

**Keywords:** phytochemicals, tannins, flavonoids, polyphenols, Geraniaceae

## Abstract

In recent years, the increase in antibiotic resistance demands searching for new compounds with antimicrobial activity. Phytochemicals found in plants offer an alternative to this problem. The genus Pelargonium contains several species; some have commercial use in traditional medicine such as *P. sinoides*, and others such as *P. peltatum* are little studied but have promising potential for various applications such as phytopharmaceuticals. In this work, we characterized the freeze-dried extracts (FDEs) of five tissues (root, stem, leaf, and two types of flowers) and the ethyl acetate fractions from leaf (Lf-EtOAc) and flower (Fwr-EtOAc) of *P. peltatum* through the analysis by thin-layer chromatography (T.L.C.), gas chromatography coupled to mass spectrometry (GC-MS), phytochemicals quantification, antioxidant capacity, and antimicrobial activity. After the first round of analysis, it was observed that the FDE-Leaf and FDE-Flower showed higher antioxidant and antimicrobial activities compared to the other FDEs, for which FDE-Leaf and FDE-Flower were fractionated and analyzed in a second round. The antioxidant activity determined by ABTS showed that Lf-EtOAc and Fwr-EtOAc had the lowest IC_50_ values with 27.15 ± 1.04 and 28.11 ± 1.3 µg/mL, respectively. The content of total polyphenols was 264.57 ± 7.73 for Lf-EtOAc and 105.39 ± 4.04 mg G.A./g FDE for Fwr-EtOAc. Regarding the content of flavonoid, Lf-EtOAc and Fw-EtOAc had the highest concentration with 34.4 ± 1.06 and 29.45 ± 1.09 mg Q.E./g FDE. In addition, the minimum inhibitory concentration (M.I.C.) of antimicrobial activity was evaluated: Lf-EtOAc and Fwr-EtOAc were effective at 31.2 µg/mL for *Staphylococcus aureus* and 62.5 µg/mL for *Salmonella enterica*, while for the *Enterococcus feacalis* strain, Fwr-EtOAc presented 31.2 µg/mL of M.I.C. According to the GC-MS analysis, the main compounds were 1,2,3-Benzenetriol (Pyrogallol), with 77.38% of relative abundance in the Lf-EtOAc and 71.24% in the Fwr-EtOAc, followed by ethyl gallate (13.10%) in the Fwr-EtOAc and (Z)-9-Octadecenamide (13.63% and 6.75%) in both Lf-EtOAc and Fwr-EtOAc, respectively.

## 1. Introduction

Plants’ primary metabolism is essential for their survival, characterized by synthesizing the necessary metabolites for their growth and functionality, such as sugars, fatty acids, and amino acids, among others. On the other hand, secondary metabolism is not essential for their survival; it is generated in response to chemical, physical, and biological factors resulting from the interaction between the plant and the environment. Secondary metabolism confers defense to biotic and abiotic stress, triggering a synthesis response of secondary metabolites that activates the plant’s defense mechanism [1,2].

It has been estimated that plants can produce more than 200,000 secondary metabolites; among them, terpenes, alkaloids, and polyphenols are the most diverse [3,4]. Polyphenols are a well-known group of compounds with antioxidant properties present in plants among the secondary metabolites. Polyphenols are present in more significant quantities in epidermal and sub-epidermal cells’ walls and vacuoles. The basic structure of polyphenols is composed of a phenyl ring with a hydroxyl group attached to it. This basic structure allows polyphenols to be classified into different subfamilies, including flavonoids and tannins [5,6,7].

Flavonoids have a 15-carbon phenyl benzopyran skeleton [5]. They are synthesized in all parts of the plant and are responsible for the pigmentation of flowers. Polyphenols protect the plant from certain insects and ultraviolet radiation [8]. Tannins are molecules with high molecular weight (500 to 20,000 Daltons), produced in seeds, roots, bark, wood, and leaves. They are characterized by an astringent taste [9] that generates protection against predators such as birds, herbivores, and some insects [5].

Tannins are classified as hydrolyzable and condensed tannins. Hydrolyzable tannins can be hydrolyzed with acids, bases, and hot water. This class of tannins is divided into gallotannins with a central structure of glucose, and ellagitannins result from the oxidation of penta-galloylglucose. Condensed tannins, also called proanthocyanidins are flavonoid oligomers with varying degrees of polymerization [9]. These metabolites are characterized by their ability to scavenge free radicals, such as reactive oxygen species (R.O.S.), reactive nitrogen species (R.N.S.), and reactive sulfur species (R.S.).

These radical species affect cells through various mechanisms, such as destabilization of cell membrane structure, interference in signaling pathways, alteration of D.N.A. integrity, disruption of homeostasis, and induction of imbalance in organisms. All these mechanisms can lead to cardiovascular and chronic degenerative diseases and some types of cancer [10].

Many patients in clinics present complications caused by microorganisms since the treatments are not as efficient as required because bacteria have developed resistance to certain antibiotics, causing complications and delays in the recovery of health in patients [11]. In fact, we are going through a stage that may become critical since antibiotics are becoming less effective and common bacterial infections that were easily cured are now more complicated to treat. These complications are due to the increasing antibiotic resistance in bacterial strains. This phenomenon worsens, and many people could risk losing their lives due to a lack of adequate and effective treatment. Therefore, the search for new active ingredients with antimicrobial activity is required, and one primary source is plants.

Plants are an excellent renewable source of chemical compounds with various properties beneficial to humans. However, plants produce a wide variety of compounds, making it difficult to identify specific compounds associated with a given property. To solve this challenge, various chromatographic techniques have been developed to separate and identify the compounds of a complex sample, such as gas chromatography coupled to mass spectrometry (GC-MS), used in several studies for the identification of different compounds with antimicrobial activity obtained through the extraction of various parts of the plant and with different types of solvents [12,13,14,15,16].

Several studies researched the composition of the aerial parts of plants by GC/MS to characterize the volatile compounds in the extracts. For example, 77 compounds were found accumulated in mint extract, within which menthol and p-mentahn3-one had the highest antimicrobial activity [17]. Some studies relate antioxidant activity and the profile of volatiles in plants [18,19,20,21]. On the other hand, there are few studies of characterization by GC/MS in the genus Pelargonium, including *P.graveolens* [21], *P. quercetorum* [18], and *P. capitatum* [22].

In traditional medicine, the use of plant extracts is common. An example of this is the standardized extract [23] from the *Pelargonium sidoides* plant, an auxiliary in treating respiratory tract complications [24]. To this extract, it is attributed antimicrobial activity [25,26], antiviral activity [27], antitumor activity [28], and antioxidant synergistic potential [29]. Antimicrobial and antiviral activities have been attributed to gallic acid, its methyl ester derivatives, and coumarins such as 5,6,7-trimethoxycoumarin and 6,8-dihydroxy-5,7-dimethoxycoumarin [25,30]. *P. sidoides* belong to the Geraniaceae family, most of which are native to Africa and comprise 11 genera, including the genus Pelargonium, which comprises more than 7500 species [31,32].

However, the diverse biological activities are not exclusive to the species *P. sidoides*; for example, antimicrobial activity has been reported against *Staphylococcus aureus* in extracts of *P. graveolens*. In this study, the antimicrobial activity of essential oil from *P. graveolens* was attributed to compounds such as β-citronellol, β-caryophyllene, and caryophyllene oxide [33,34]. In the case of *P. peltatum*, biological activity against *Streptococcus mutans* and *Streptococcus tanguinis* has also been reported. Nevertheless, more studies are needed to demonstrate the potential of the compounds present in *P. peltatum*, both in their antimicrobial properties and antioxidant potential. Therefore, the purpose of this research was to perform a phytochemical, antioxidant, and GC-MS profile of hydroalcoholic extracts of root, stem, leaf, and flower of *P. peltatum*, seeking to compare the differences in the concentration of antioxidant compounds among the different tissues and to evaluate which of the extracts obtained had the highest antioxidant and antimicrobial activity and the possible semi-volatile compounds related to them.

## 2. Results

### 2.1. Yield and Solubility of Freeze-Dried Extract from P. peltatum

Table 1 shows the dry weight of each part of the plant and the weight obtained from the freeze-dried extracts (FDE). The freeze-dried flower extract (FDE-Bicolour) showed the highest recovery with a 28.47% yield, while the freeze-dried stem extract (FDE-Stem) showed the lowest with a 6.65% yield. From now on, we will refer to the freeze-dried extracts as FDE-Root, FDE-Stem, FDE-Leaf, FDE-Flower, and FDE-Bicolour. The lyophilized extracts were subjected to solubility tests. Most of the extracts were soluble in polar solvents, as shown in Supplementary Table S1.

### 2.2. Phytochemical Profiling by High-Performance Thin Layer Chromatography

The FDEs of *P. peltatum* were analyzed by thin-layer chromatography (T.L.C.) using a mobile phase of water: methanol: ethyl acetate (10:14:76) for their separation. The chromatoplates were developed using specific reagents for each group of compounds and exposure to ultraviolet light at two wavelengths (254 and 366 nm). For the development of tannins, we used a 1% ferric chloride solution; the chromatoplates showed the presence of bands in all the FDE. In particular, two bands (with RF = 0.015 and RF = 0.036) were present in all extracts, suggesting that the compounds found in these retention factors are common in all plant tissues. Similarly, a 1% aluminum chloride developer was used for flavonoids. In this case, all extracts presented bands reactive to this solution, where FDE-Flower showed the highest complexity of banding pattern (13 bands). Table 2 summarizes the retention factors (R.F.) for the bands detected in each experiment. In the case of the chromatoplates visualized at 254 nm, very few bands were observed in only two extracts, while the banding patterns visualized at a wavelength of 366 nm presented complex banding patterns in all extracts (see Supplementary Figure S1). We were unable to detect compounds with the alkaloid developer.

### 2.3. Antioxidant Activity of Freeze-Dried Extract from P. peltatum

The antioxidant capacity of the FDEs samples were evaluated using the DPPH, ABTS, and FRAP methodologies. The results are shown in Table 3. In these experiments, we evaluated different concentrations of the FDEs (20–500 µg/mL), and the results were expressed as IC_50_ (µg/mL) for ABTS and DPPH. FRAP measurements are reported in µM Trolox equivalent (T.E.).

When the DPPH technique determined the antioxidant activity, the FDE-Leaf showed the lowest IC_50_ value with 46.32 ± 1.84 µg/mL, followed by FDE-flower with 48.9 ± 3.36 µg/mL. Both values did not show significant differences between them. In comparison, FDE-Root showed the highest IC_50_ value with 77.47 ± 6.92 µg/mL, followed by FDE-Stem with 75.02 ± 10.57 µg/mL. Similarly, the results obtained by the ABTS method showed that FDE-Flower and FDE-Leaf had the lowest IC_50_ values with 103.9 ± 19.29 and 132.5 ± 19.15 µg/mL, respectively. In contrast, FDE-Stem showed the highest IC_50_ value with 293.40 ± 24.75 µg/mL. Measurements performed with FRAP methodology showed that FDE-Leaf has the highest antioxidant activity with 274.00 ± 3.70 µM T.E. followed by FDE-Flower with 258.70 ± 2.46 µM T.E. This time, a significant difference between these two samples was observed. Instead, FDE-Root showed the lowest value with 44.03 ± 3.79 µM T.E. Together these results indicate that FDE-Leaf and FDE-Flower have the highest, while FDE-Root and FDE-Stem have the lowest antioxidant capacity.

### 2.4. Quantification of Polyphenols, Tannins, and Flavonoids in FDEs from P. peltatum

**Figure 1:**
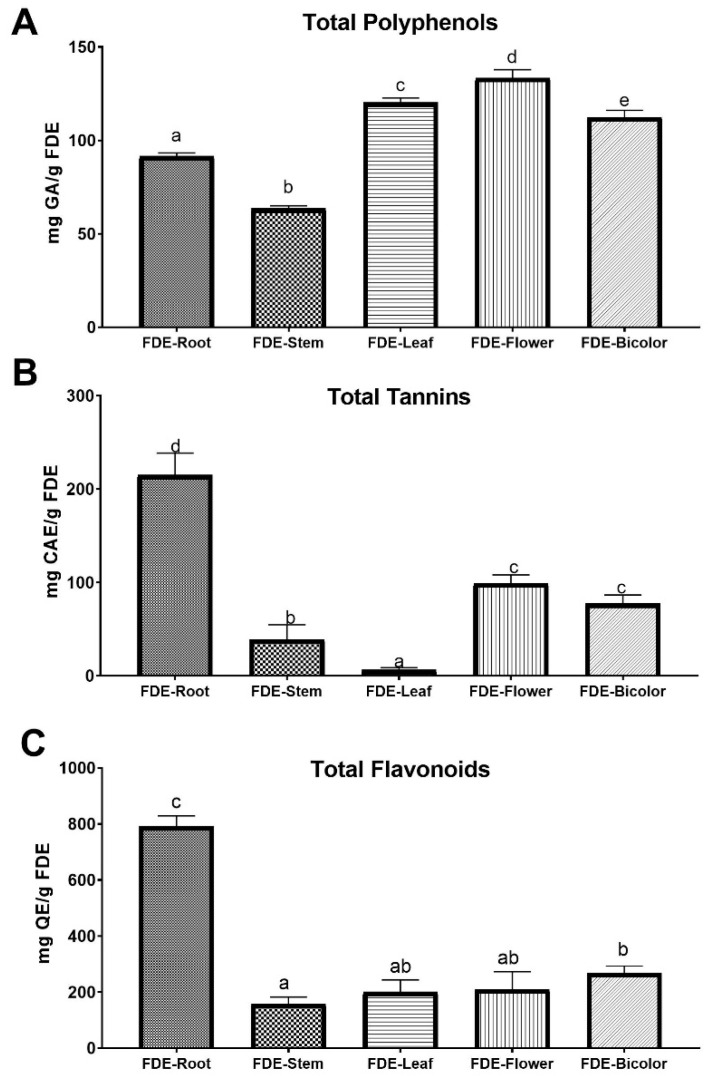
Quantification of secondary metabolites from *P. peltatum* FDE. (**A**) total polyphenols, (**B**) tannins, and (**C**) flavonoids in hydroalcoholic extract of *P. peltatum*. The histogram shows the values of the means ± S.D. of three independent experiments and the Tukey L.S.D. test showing homogeneous groups with a *p* < 0.005. Different lowercase letters (a, b, c, d and e) on the top of columns represent significant difference (*p* < 0.05).

Total polyphenol content was determined spectrophotometrically according to the Folin–Ciocalteu methodology. The results were expressed as mg of gallic acid (G.A.) equivalents per g of FDE. The highest concentration of polyphenols was found in FDE-Flower and FDE-Leaf with 133.36 ± 4.4 and 120 ± 2.4 mg G.A. equivalents/g of FDE, respectively. In comparison, FDE-Stem was the sample with the lowest concentration of polyphenols with 63.53 ± 1.4 mg G.A. equivalents/g of FDE. The graphed values can be seen in Figure 1A.

Tannin concentration was determined by the vanillin method. The results were expressed in mg of catechin equivalents (Q.A.)/g of dry extract. The FDE-Root had the highest tannin content with 214.99 ± 21.3 mg Q.A. equivalents/g of FDE, being almost twice as high as the FDE-Flower extracts with 99.02 ± 8.2 and the FDE-Bicolour with 77.14 ± 8.2 mg Q.A. equivalents/g of FDE. In contrast, the FDE-Leaf had the lowest tannin concentration with 6.23 ± 7.5 mg Q.A. equivalents/g of FDE. These values are plotted in Figure 1B, where the significant differences between the samples can be observed with a confidence level of 95% using the ANOVA and Tukey test.

The flavonoid content was performed using the aluminum trichloride method. Results were expressed as mg quercetin equivalents (Q.E.)/g of FDE. The FDE-Root showed the highest amount of flavonoids with 743.5 ± 36.7 mg Q.E. equivalents/g of FDE (Figure 1C) compared with the rest of the extracts, which showed about three times less. This time, the FDE-Stem was the sample with the lowest flavonoid content with 157.58 ± 24.6 mg Q.E. equivalents/g of FDE.

### 2.5. Antimicrobial Activity of Freeze-Dried Extract from P. peltatum

The FDEs of *P. peltatum* were tested to inhibit bacterial growth using four strains: *Staphylococcus aureus*, *Enterococcus faecalis* (Gram + bacteria), *Salmonella enterica*, and *Serratia marcescens* (Gram-bacteria). The FDEs were diluted with sterile water to a 5 mg/mL concentration and then assayed in the agar-well diffusion method. The FDEs exhibited microbial inhibitory activity in all strains tested (Figure S2). However, FDE-Leaf, FDE-bicolour, and FDE-Flower presented generally more considerable inhibition halos than FDE-Stem and FDE-root. Gram-positive strains were more susceptible to inhibition by FDEs than gram-negative strains. Notably, *E. faecalis* was highly susceptible to growth inhibition with FDE-Leaf. Generally, the FDEs with the highest activity are also those with the highest presence of polyphenols. The inhibition zones generated by FDEs against *S. aureus*, *E. faecalis*, *S. enterica*, and *S. marcescens* are presented in Supplementary Table S2.

### 2.6. GC-MS Analysis of FDEs from P. peltatum

**Figure 2:**
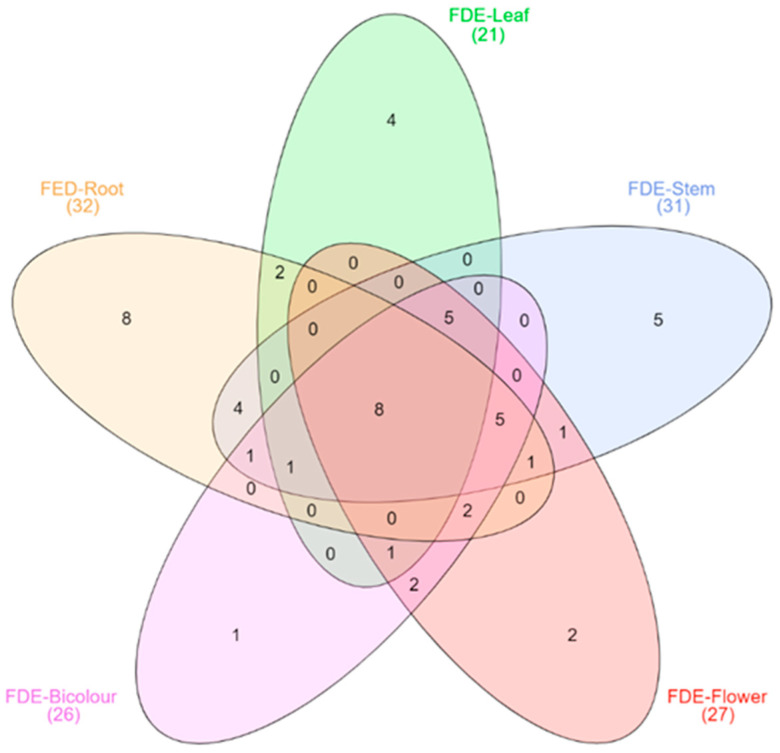
Venn diagram showing the volatile and semi-volatile compounds unique and shared between the FDEs of *Pelargonium peltatum*.

In order to know the chemical composition of the five extracts, we decided to perform a screening of volatile and semi-volatile compounds by gas chromatography coupled with mass spectrometry (GC-MS). A total of 53 compounds were identified by GC-MS analysis of polar extracts (Table 4). These compounds were esters, carboxylic acids, aldehydes, benzene derivatives, phenolic compounds, aromatic compounds, and terpenes. The FDE-Root had the most diverse chemical composition with 32 compounds, of which eight were unique, while the leaf extract was the least diverse with 21 compounds, of which four were unique, and only eight compounds are shared among all five FDEs (Figure 2).

The ethyl gallate and 1,2,3-Benzenetriol, also known as pyrogallol, are the most abundant compounds in the leaf, stem, flower, and bicolour extracts, while in the root, tridecanoic acid and 2,3-dihydro-3,5-dihydroxy-6-methyl-4*H*-pyran-4-one are the most abundant. This difference in chemical composition could be related to the nature of the samples, since the root is the underground part, while the leaf, stem, and flower are the aerial part of the plant.

### 2.7. Kupchan Partitioning of FDE-Flower and FDE-Leaf

The GC-MS analysis of the FDEs yielded important information about their chemical composition. However, the number of identified compounds was relatively high, and the data obtained in the antimicrobial activity assay showed that this effect was observed at high concentrations, suggesting that the compound(s) associated with this activity were diluted in the samples. In order to define these compounds, the fractionation of the FDEs was carried out using solvents with different polarities. In this case, we decided to work exclusively with the flower and leaf samples because, in the antioxidant and antimicrobial activity experiments, both FDEs presented the highest activity in both analyses. Likewise, the FDE-flower and FDE-bicolour were worked together since the GC-MS data showed that both shared 85% of the compounds. Four solvents were used: hexane (Hx), chloroform (Chl), ethyl acetate (EtOAc), and methanol (MeOH), using a Kupchan partition sequence.

The FDE-Flower and FDE-Leaf samples were subjected to solvent partitioning. The yield with the Hx fraction was practically null, so it will no longer be reported during this study. On the other hand, we obtained sufficient yields for our experiments with the FDE partitioned with Chl, EtOAc, and MeOH.

### 2.8. Antioxidant Activity of FDE-Flower and FDE-Leaf Partitioned Extracts

We analyzed the fractions extracted with the solvents using the three aforementioned antioxidant capacity measurement techniques. In all three methods used, the fractions with the highest antioxidant activity were those of FDE-Leaf and FDE-Flower partitioned with EtOAc (Lf-EtOAc and Fwr-EtOAc, respectively). The antioxidant activity values obtained with ABTS for the fractions Lf-EtOAc and Fwr-EtOAc had the highest Trolox equivalents per gram of lyophilisate extract (mEq Trolox/g FDE) being 2136.90 ± 68.79 and 1964.63 ± 48.41 mEq Trolox/g FDE. On the other hand, the fractions obtained by chloroform extraction for FDE-Leaf (Lf-Chl) and FDE-Flower (Fwr-Chl) showed significant differences in their antioxidant activity measured by ABTS. The activity of Lf-Chl was comparable to that of Lf-EtOAc and Fwr-EtOAc with a value of 2118.20 ± 209.55 mEq Trolox/g FDE.

Fwr-Chl had low activity compared to Lf-Chl (118.28 ± 1.22 mEq Trolox/g FDE). The fractions partitioned with MeOH, FDE-Leaf (Lf-MeOH), and FDE-Flower (Fwr-MeOH) did not present significant differences and had relatively low values concerning the EtOAc fractions, being 175.10 ± 19.77 and 121.27 ± 27.15 mEq Trolox/g FDE, for Lf-MeOH and Fwr-MeOH, respectively.

**Figure 3:**
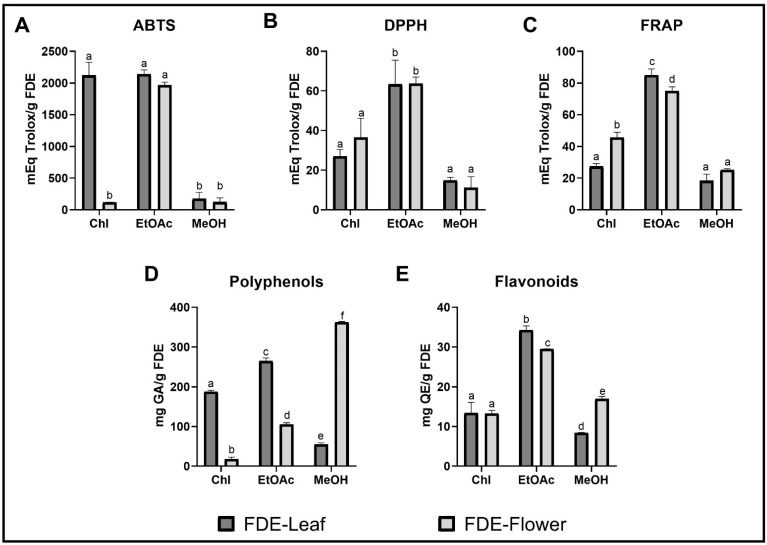
Antioxidant activity and quantification of secondary metabolites in the different fractions of *P. peltatum*. The antioxidant activity was determined by (**A**) ABTS, (**B**) DPPH, and (**C**) FRAP. The quantified secondary metabolites were (**D**) polyphenols and (**E**) flavonoids. Chl: Chloroform fraction, EtOAc: ethyl acetate fraction, MeOH: methanol fraction. The histogram shows the values of the means ± S.D. of three independent experiments and the Tukey L.S.D. test showing homogeneous groups with a *p* < 0.005. Different lowercase letters (a, b, c, d and e) on the top of columns represent significant difference (*p* < 0.05).

Regarding the other antioxidant capacity measurement methodologies, the trend indicated that for both methodologies (DPPH and FRAP), the fractions with the highest antioxidant activity were Lf-EtOAc and Fwr-EtOAc. The graphs of the reported antioxidant activities can be seen in Figure 3A–C. We decided to evaluate also the content of total phenols and flavonoids in these fractions. Interestingly, total polyphenols (Figure 3D), presented the highest values in the fractions Lf-EtOAc, Lf-Chl, and Fwr-MeOH, with 264.57 ± 7.73, 187.46 ± 3.86, and 362.33 ± 12.07 mg G.A./g FDE. Likewise, flavonoid content (Figure 3E) was quantified. On this occasion, Lf-EtOAc and Fw-EtOAc fractions had the highest concentration with 34.4 ± 1.06 and 29.45 ± 1.09 mg Q.E./g FDE. Tannins were not detectable in the measurements performed for all fractions. These results indicated that the Lf-EtOAc and Fwr-EtOAc fractions had the best profiles of antioxidant capacity, polyphenol, and flavonoid content.

### 2.9. M.I.C. of Lf-EtOAc and Fwr-EtOAc Fractions against Pathogenic Bacteria

Once the fractions antioxidant activities and phytochemical content were verified, we decided to evaluate the minimum inhibitory concentration (M.I.C.) against the pathogenic bacteria evaluated in the experiment with the FDEs. However, this time, we took advantage of the decrease in the complexity of the compound mixtures achieved by solvent partitioning. Among the fractions evaluated, it was clear that Lf-EtOAc and Fwr-EtOAc were by far the ones with the best minimum inhibitory concentration, reaching 31.2 µg/mL for *S. aureus* and 62.5 µg/mL for *S. enterica*. While for *E. faecalis* strain, the Fwr-EtOAc fraction presented 31.2 µg/mL of M.I.C. In contrast, the Lf-EtOAc fraction was twice as high at 62.5 µg/mL. It should be noted that the antimicrobial effect against *S. marcescens* was greatly diminished since both EtOAc fractions presented a M.I.C. of 500 µg/mL. The rest of the fractions presented high M.I.C. values; therefore, they were considered ineffective for antimicrobial inhibition. The data are summarized in Table 5.

### 2.10. GC-MS Analysis and IC_50_ of Lf-EtOAC and Fwr-EtOAC Fractions

This time, nine compounds were identified in the leaf fraction and 12 compounds in the Lf-EtOAc fraction. The main compounds were 1,2,3-Benzenetriol (Pyrogallol), with 77.38% of relative abundance in the Lf-EtOAc’s fraction and 71.24% in the Fwr-EtOAc fraction, followed by ethyl gallate (13.10%) in the Fwr-EtOAc fraction and (*Z*)-9-Octadecenamide (13.63% and 6.75%) in both fractions, Lf-EtOAc, and Fwr-EtOAc, respectively (Table 6). Additionally, we calculated the IC_50_ values for the Lf-EtOAc and Fwr-EtOAc fractions. The IC_50_ obtained for the Lf-EtOAc by ABTS was 27.15 ± 1.04 and 33.66 ± 1.50 µg/mL with the DPPH technique. On the other hand, values of 28.11 ± 1.3 and 52.69 ± 1.6 µg/mL of IC_50_ were obtained for the Fw-EtOAc fraction by ABTS and DPPH technique, respectively.

## 3. Discussion

The extracts of root, stem, leaf, flower, and bicolour flower of *Pelargonium peltatum* were obtained by hydroalcoholic maceration at 70%, and the chromatographic profile was analyzed. The phytochemical profile with the highest complexity was FDE-flower, which has been mentioned in phytochemical studies of other species belonging to the *Pelargonium* genus. However, it is essential to emphasize that species belonging to the *Pelargonium* genus, such as *P. sidoides*, have a high demand because of their use as a constituent in the formulation of phytopharmaceuticals. Due to its exploitation and the unfavorable factors in its habitat, *P. sidoides* is experiencing a decrease in its natural population and a deterioration of its biological niche. Thus, this research on the phytochemical and antioxidant profile of *P. peltatum* contributes to producing information to offer an alternative in the future as a possible source of bioactive components [35].

Polyphenols are compounds whose basic structure is hydroxyl groups attached to a phenyl ring. One of its qualities is its antioxidant activity, and in this study, a high content was detected in the FDE-Flower and FDE-Leaf. In work by Mona M. and co-workers [36] showed high polyphenol content in the extract of *P. sidoides*, as did Malek Ennaifer in *P. graveolens*, [37] and Gökçe Şeker Karatoprak and co-workers [38] in *P. endlicherianum*, indicating that a high content of polyphenolic compounds is a characteristic in common between the different species of the genus *Pelargonium*. Polyphenols are highly relevant for their beneficial impact on health since they inhibit factors that can cause cardiovascular diseases (v.g. hydroxytyrosol, quercetin, and resveratrol), give protection against different types of cancer (v.g. apigenin, quercetin, curcumin, resveratrol, EGCG, and kaempferol), and help inhibit inflammatory processes in chronic degenerative diseases (e.g., apigenin, epigallocatechin gallate, ellagic acid, and rutin) that have a high incidence in the world population [39,40]. The profiles obtained by GC/MS found that one of the major components of the polyphenols is pyrogallol, among others such as catechol, eugenol, vanillic acid, methyl, and ethyl gallate.

The antioxidant activities for the FDEs presented values of low antioxidant activity measured by IC_50_, except for those reported for FDE-Flower and FDE-Leaf, whose values ranged between (46.32–48.90 µg/mL for ABTS and 132.50–103.90 µg/mL for DPPH). IC_50_ values for extracts and essential oils of *P. graveolens* have been reported in the literature ranging from 711–1280 μg/mL for oils and 12.24–44.24 μg/mL for extracts measured by DPPH [21]. This places our results below the values obtained for oils and above those obtained for extracts. In comparison with other species, the IC_50_ values reported for *Nepeta melissifolia* (IC_50_ = 13.5 ± 0.4 μg/mL) [41] and *Mentha pulegium* (IC_50_ = 69.60 ± 1.72 μg/mL) [42] were in comparison to our measurements by DPPH much higher in their IC_50_. On the other hand, once the extracts were fractionated, the Lf-EtOAc and Fwr-EtOAc produced IC_50_ values ranging from 27.15–33.66 µg/mL for ABTS and 28.11–52.69 µg/mL for DPPH. Those values are closer to those reported in extracts of other plants with broad antioxidant power [43,44] and even relatively close to references in antioxidants of pure compounds such as BHT (18.5 ± 0.4 ug/mL) [45] but still distant from classical antioxidants such as ascorbic acid (3.9 ± 0.3 μg/mL) [46].

At least two antimicrobial compounds were detected in the Lf-EtOAc and Fwr-EtOAc fractions in the GC-MS analysis, the first one is pyrogallol (1,2,3-trihydroxybenzene) which has been reported several times as an antimicrobial and antioxidant compound. In the work reported by [47], they demonstrate that a dimer of this compound can produce a MIC value for *Staphylococcus aureus* and *Escherichia coli* of (8 μg/mL) and in its non-polymerized form of 512 and 256 μg/mL, respectively. This study was performed with a pure compound, while in our analysis a MIC value of 31.2 μg/mL was found in the analyzed fractions of EtOAc against *S. aureus* and of the Fwr-EtOAc fraction against *E. faecalis*. In fact, pyrogallol is classified as an allelochemical [48,49] and other polyphenolic compounds in conjunction with pyrogallol have been evaluated to potentiate the effect of known antibiotics [50]. It is important to mention, that there are new studies regarding the possibilities of the use of pyrogallol, in material sciences, and due to the complexity of its synthesis, extractions from the plant kingdom are still a suitable option for its isolation [51]. The other compound found in our study from acetate fractions is ethyl gallate. This compound has been reported with a MIC of 0.24 and 0.48 mg/mL in extracts of *R. crenulata*, against *E. coli* and *S. aureus* strains. Although these MIC concentrations are very high compared to the highest MIC values obtained in this study for acetate fractions (500 ug/mL), other reports have indicated the potential of its antioxidant and antimicrobial activity [52,53,54,55,56,57].

Interestingly, our analysis showed the presence of (*Z*)-9-Octadecenamide, in the acetate fractions, this compound has been known by the common name oleamide and has neurological activities, in fact, it is a lipid-like molecule that induces sleep [58], but it also has activities that regulate memory processes and antinociception [59]. Other reports have associated it with an algaecidal and fungicidal agent [60].

Finally, in the food industry, many molecules of synthetic origin, such as BHT and its derivatives, are used to improve processed foods’ quality and shelf life; however, these types of molecules can have adverse effects, leading to the development of tumor tissues [61]. That is why it is crucial to identify naturally occurring molecules with antioxidant activity. In this work, we detected 53 different compounds by GC/MS in the five FDE extracts, including furan derivatives, furfurans, polyphenols, fatty acids, and various esters of gallic acid. Interestingly, the fractions obtained by partitioning with ethyl acetate in flower and leaf reported excellent antimicrobial and antioxidant activities. Overall, the results obtained in this research suggest that *P. peltatum* is an excellent option for further studies, elucidating the compounds involved in the significant antioxidant capacity values and the promising potential of antimicrobial activities.

## 4. Materials and Methods

### 4.1. Reagents

All chemicals used were of analytical grade and were used as received without any further purification. All solutions were prepared with deionized water.

### 4.2. Preparation of Extracts

#### 4.2.1. Harvesting and Identification of *P. peltatum*

Plant material was obtained from various nurseries in the city of Guadalajara Jalisco between February and June 2020, one of the specimens was used for taxonomic identification by Dr. Liberato Portillo of the Centro Universitario de Ciencias Biológicas y Agropecuarias (CUCBA) of the University of Guadalajara and stored in the IBUG (Herbarium Luz María Villareal de Puga) generating the ID: SIST-TRA-2020-2.

#### 4.2.2. Cleaning and Drying of Collected Material

Plants were separated by their root, stem, leaf, and flower sections (in the case of flowers, they were subdivided into flowers of a single color and bicolored flowers), and then the general dirt was removed with potable water and rinsed with injectable water. The plant material was dried at room temperature in the absence of light, and the help of printing paper was changed every 24 h to avoid the accumulation of humidity.

#### 4.2.3. Extracts Preparation

The dried plant material of root (158 g), stem (290 g), leaf (200 g), flower (150), and bicolor (144 g) was deposited in amber flasks, and 2 L of 70% ethanol was added to each sample. These flasks were kept under agitation for 15 days. Subsequently, the extracts obtained were filtered through Whatman’s paper three times. On each occasion, the filter was exchanged to remove the separated solids.

#### 4.2.4. Concentration and Lyophilization of Extracts

The extracts were concentrated by rotary evaporation to obtain the aqueous phase in a Buchi R-200 equipment. The concentrated extracts were then placed in freeze-drying bottles, and with the help of acetone and CO_2_, the samples were frozen and immediately placed in a LABCONCO freeze-dryer for 24 h at −51 °C with a pressure of 0.090 mbar, protected from light. Once lyophilization was completed, the extracts were aliquoted in a laminar flow hood and stored under refrigeration at 4 °C until use.

### 4.3. Solubility Test

Next, 1 mg of the lyophilized material of the five hydroalcoholic extracts of *P. peltatum* were deposited in test tubes, and subsequently, the solubility was evaluated by adding 1 mL of different solvents (polar and non-polar). The results were expressed with the legend: (−) not soluble, (+) soluble (++), and very soluble.

### 4.4. Phytochemical Profiling by High-Performance Thin Layer Chromatography

The lyophilizates of each tissue were resuspended with distilled water to a concentration of 10 mg/mL. Then, 15 µL of these solutions were deposited in bands with a length of 8 mm each with a Camag syringe of 100 µL on aluminum support plates coated with silica gel 60F254 (20 × 10 cm) by an autosampler Camag Linomat 5.

The plates were then placed in a glass chamber previously saturated (30 min) with the mobile phase: water–methanol–ethyl acetate (10:14:76 v:v, respectively) [23]. Once the plates were dried, they were visualized in a U.V. chamber at two wavelengths of 254 and 366 nm. Additionally, T.L.C. plates were developed with specific solutions for each group of compounds: ferric chloride solution for tannin determination, trichloroacetic acid solution for glycosides, Dragendorff’s reaction for alkaloids, and aluminum chloride solution for flavonoids.

The retention factor was calculated using the following equation:

(1) $$Rf=\frac{Distance\;the\;band}{Distance\;of\;the\;solvent}$$

### 4.5. Antioxidant Activity

The antioxidant activity of the hydroalcoholic extract of *P.* *peltatum* was determined by DPPH, ABTS, and FRAP techniques and dilutions of the freeze-dried extracts of *P. peltatum* were prepared from 13 to 1000 µg/mL. These samples were used for antioxidant and phytochemical determinations. The half-maximal inhibitory concentration (IC_50_) was calculated with the statistical program Graph pad version 9 with simple ANOVA analysis.

#### 4.5.1. 2,2-diphenyl-1-picrylhydrazyl Hydroxyl Radical (DPPH) Activity

The DPPH radical scavenging assay was carried out according to the methodology of Brand–Williams [62] with minor modifications. Briefly, the working solution of DPPH was prepared at 0.04% (*w*/*v*) in methanol and protected from light. In a microplate, 20 µL of the sample and 280 µL of the working solution were added in triplicate. The mixtures were incubated for 30 min at room temperature under the dark, and the absorbance was measured at 515 nm. Trolox was used as a standard with a curve between 0 and 1280 µg/mL. The results were expressed as percentage inhibition using the following equation:

(2) $$I\left(\%\right)=1-\frac{AB_{control}-AB_{sample}}{AB_{control}}\left(100\right)$$

#### 4.5.2. 2,2′-azino-bis-(3-ethylbenzothiazolin-6-sulfonic acid) (ABTS) Radical Scavenging Activity

The ABTS radical scavenging assay was performed by the technique reported by Li et al. [63] with minor modifications. A stock solution of 7 mM ABTS was prepared with 4 mM ammonium persulfate (P.S.A.) in distilled water. The solution was kept at 4 °C for 12 h to allow radical formation. The working solution was then prepared by adjusting the optical density to 0.7 ± 0.02 units at 750 nm. In a microplate, 280 µL of the working solution was placed in triplicate with 20 µL of each sample. The reaction was monitored at 750 nm for 30 min. Trolox was used as a standard with a curve between 0 and 400 µg/mL. The results were expressed as mg Trolox equivalents per g of dry extract. When indicated IC_50_ was determined.

#### 4.5.3. Ferric Ion Reducing Activity (FRAP)

The ferric reducing antioxidant power was evaluated by the method described by Benzie et al. [64]. A working reagent was prepared by mixing: 2.5 mL of 2,4,6-Tris(2-pyridyl)-s-triazine (TPTZ) at 10 mM, 2.5 mL of Ferric chloride (FeCl3) at 20 mM, and 25 mL of 0.1 M acetate buffer at pH 3.7. The reaction was carried out in a microplate by placing 40 µL of the sample, followed by 40 µL of acetate buffer pH 4.7 with 150 µL of working reagent. Readings were taken at 595 nm after 30 min, and each sample was analyzed by triplicate. Trolox was used as a standard with a curve between 0 and 400 µg/mL. The results were expressed as mg of Trolox equivalents per g of dry extract.

#### 4.5.4. Total Polyphenol Content

Total polyphenol content was determined spectrophotometrically, using Folin–Ciocalteu reagent and gallic acid as standard, following the procedure reported by Singleton [65]. To 125 µL of each sample, 750 µL of distilled water and 62.5 µL of Folin–Ciocalteu reagent were added; after 5 min of incubation in the dark, 250 µL of distilled water and 187.5 µL of 20% (*w*/*v*) Na_2_CO_3_ were added, and the mixture was incubated for 2 h in the dark. After that time, 200 µL of each reaction was placed in triplicate in microplates, and absorbance was measured at 750 nm. The calibration curve was made in the range 0 to 100 µg/mL of gallic acid. The results were expressed as mg of gallic acid equivalents per g of dry extract.

#### 4.5.5. Total Tannin Content

The quantification of tannins was determined by the Broadhurst and Jones method [66]. A 4% vanillin solution in methanol (4 g/100 mL) was prepared. Then in an Eppendorf tube, 1 mL of the vanillin solution, 133 µL of extract, and 500 µL of concentrated hydrochloric acid were added. Solutions were mixed and incubated for 15 min and then 200 µL of the solution was dispensed by triplicate in a 96-well plate. Readings were taken at 500 nm of absorbance. A curve of catechin was made from 0 to 100 µg/mL. The results were expressed as mg of catechin equivalents per g of dry extract.

#### 4.5.6. Total Flavonoid Content

The determination was performed by the aluminum trichloride (AlCl_3_) method, as reported by Zhishen et al. [67]. To 125 µL of each extract, 500 µL of distilled water, and 37.5 µL of 5% (*w*/*v*) sodium nitrite (NaNO_2_) was added and incubated at room temperature for 5 min. After this time, 37.5 µL of 10% (*w*/*v*) AlCl_3_ was added and incubated for 6 min. Finally, 300 µL of distilled water and 250 µL of 1 M sodium hydroxide (NaOH) were added. After 15 min, the mixture was homogenized, and 200 µL were transferred in triplicate to a microplate. Finally, the absorbance at 510 nm was determined. A quercetin standard curve was used in a range of concentrations between 0 and 1000 µg/mL. The results were expressed as µg quercetin equivalents per g of dry extract.

### 4.6. Antimicrobial Activity

Gram-negative (*Salmonella enterica/Serratia marcescens*) and Gram-positive (*Enterococcus faecalis*/*Staphylococcus aureus*) bacterial strains were used for the antimicrobial assays. Then, 5 mL of inoculum of each bacterial strain grown on Tripticasein Broth (T.S.B.) medium were placed at 37 °C in an incubator until reaching an optical density at 600 nm of 0.5 ± 0.02 units. Subsequently, 200 µL of these cultures were inoculated into 25 mL of molten T.S.B. agar. This agar was placed in Petri dishes and kept at room temperature until completely solidified. Each plate had 0.8 cm diameter wells, and each well was filled with 50 µL of each sample at a concentration of 5 mg/mL. As a positive control, 50 µL of kanamycin at 35 µg/mL was placed in the center. The plates were kept at 4 °C for 16 h to allow diffusion of the samples in agar. Subsequently, the plates were incubated at 37 °C for 36 h, and the inhibition halo was observed.

### 4.7. Gas Chromatography—Mass Spectrometry Analysis

The analysis of volatile and semi-volatile compounds of *P. peltatum* extracts was carried out with an Agilent 7890B gas chromatograph (G.C.), coupled with an Agilent 5977A mass selective detector (M.S.D.) (Agilent Technologies, Inc., Santa Clara, CA, USA), which used helium as the carrier gas with a flow of 1 mL/min. Then 20 mg of the samples were weighed and dissolved in 1 mL of HPLC-grade methanol, then filtered with a 45 μm PTFE microfilter, and 1 μL was injected into the G.C. inlet at 240 °C in splitless mode. The compounds were separated on a HP-5ms Ultra Inert capillary column (30 m × 250 μm × 0.25 μm), using the following G.C. oven temperature program: 3 min at 40 °C up to 280 °C during 2 min at 10 °C/min. The MS used electron ionization energy of 70 eV, scanning range of 30–550 uma, scan rate of 13.8 spectra/s, solvent delay of 3 min, ionization chamber temperature of 200 °C, and transfer line temperature of 250 °C. Data were processed with MassHunter Workstation software (Agilent Technologies, Inc., Santa Clara, CA, USA). Compounds were identified based on their mass spectra fragmentation patterns with the spectral database of the National Institute of Standards and Technology (NIST) and by comparing their Kovats indices (K.I.), calculated in relation to the retention times of a series of alkanes (C_7_–C_40_) using the calculator described by [68]. The relative quantities of each compound were expressed as the area percentage.

### 4.8. Kupchan Partitioning and Fractionation

The fractionation of the FDE-Leaf and Flower was performed by Emran et al.’s method [69] with minor modifications, 5 g of each extract were resuspended in 200 mL of ethanol 70%, then the solution was divided into four parts with 200 mL of different organic solvents varying its polarity (hexane, chloroform, ethyl acetate, and methanol). These new solutions were put under agitation for 10 min, and poured into a decantation funnel for the separation of their phases. Each found fraction was concentrated by the use of a rotary evaporator (Buchi-R200 Alemania) to remove the solvents. Once the fractions were concentrated, they were taken into a LABCONCO freeze-dryer for 24 h at −51 °C with a pressure of 0.090 mbar, and stored at 4 °C until further use.

### 4.9. Minimum Inhibitory Concentration

The minimum inhibitory concentration (MIC) was determined with a 96-well plate microdilution method based on resazurin (Elshikh et al., 2016). In a 96-well plate, 300 µL of the sample were placed by triplicate at 1 mg/mL dissolved in LB medium with DMSO at 2% (*v*/*v*), from this sample serial dilutions were made with 150 µL of the previous concentration and 150 µL of fresh medium until reaching a final concentration of 15.62 µg/mL. Subsequently, 15 µL of culture at 1 × 10^6^ CFU/mL of the aforementioned bacteria was added. The mix was incubated at 37 °C for 18 h. After this period, 30 µL of resazurin 0.015% was added to each well, and the plates were incubated for 1 h at 37 °C. The MIC was the lower concentration with no color change of resazurin, at the lowest concentration in which the color change of resazurin did not occur.

### 4.10. Statistical Analysis

One-way analysis of variance (ANOVA) was employed for analyzing the data and the mean difference between given treatments was intended for significance test at *p* > 0.05 level by Tukey’s HSD. The software GraphPad Prism 9 was used to statistical analysis.

## Figures and Tables

**Table 1:** Freeze-dried extract yields of *P. peltatum*.

Sample	Dry Weight (g)	Lyophilized Weight (g)	Yield (%)
FDE-Root	150	18.16	12.10
FDE-Stem	290	19.3	6.65
FDE-Leaf	200	46.04	23.01
FDE-Flower	158	37.22	23.55
FDE-Bicolour	152	43.24	28.47

**Table 2:** Retention factors (R.F.) revealed FDEs for tannins and flavonoids.

Compounds	FDE-Root	FDE-Stem	FDE-Leaf	FDE-Flower	FDE-Flower (Bicolour)
			0.015	0.015	0.015
		0.015	0.036	0.036	0.036
Tannins	0.015	0.036	0.64	0.64	0.64
	0.036	0.83	0.66	0.82	0.82
			0.85		
				0.07	
				0.21	0.21
				0.26	0.26
			0.07	0.27	0.32
			0.08	0.30	0.34
	0.07	0.07	0.36	0.4	0.43
Flavonoids	0.10	0.72	0.52	0.42	0.45
	0.73	0.73	0.54	0.45	0.56
		0.87	0.69	0.53	0.65
			0.78	0.55	0.7
				0.64	
				0.67	
				0.76	

**Table 3:** Antioxidant activity and IC_50_ of FDE from *P. peltatum*.

Sample	ABTS IC_50_ (µg/mL)	DPPH IC_50_ (µg/mL)	FRAP µM TE
FDE-Root	77.47 ± 6.92 ^b^	209.50 ± 26.20 ^b^	44.03 ± 3.79 ^a^
FDE-Stem	75.02 ± 10.57 ^b^	293.40 ± 24.75 ^c^	120.08 ± 3.79 ^b^
FDE-Leaf	46.32 ± 1.84 ^a^	132.50 ± 19.15 ^a^	274.00 ± 3.70 ^d^
FDE-Flower	48.90 ± 3.36 ^a^	103.90 ± 19.20 ^a^	258.70 ± 2.46 ^c^
FDE-Bicolour	55.36 ± 1.96 ^a^	215.60 ± 21.05 ^b^	249.29 ± 1.19 ^c^

Average of three replicates ± standard deviations. The letters in superscript denote the significant difference between samples at 95% confidence determined with ANOVA and Tukey’s test. Comparisons were made between the different extracts within the same group of methodology.

**Table 4:** Volatile and semi-volatile compounds identified in FDEs of *Pelargonium peltatum* by GC-MS.

				**% Area**						
**#**	**Name**	**MF**	**MW**	**FDE-Roots**	**FDE-** **Leaf**	**FDE-** **Stem**	**FDE-Flower**	**FDE-Bicolour**	**KI_exp_ ^(1)^**	**KI_lit_ ^(2)^**
1	2-Hydroxy-propanoic acid, ethyl ester	C_5_H_10_O_3_	118.1			4.11			821.7	821
2	Furfural	C_5_H_4_O_2_	96.0	0.41			0.14	0.15	856.5	852
3	2-Furanmethanol	C_5_H_6_O_2_	98.0	0.27		0.09			874.7	875
4	Cyclopent-4-ene-1,3-dione	C_5_H_4_O_2_	96.0	0.10					894.5	880
5	2-Acetylfuran	C_6_H_6_O_2_	110.0	0.56	0.05	0.28	0.26	0.31	921.4	921
6	5-Methyl-2-furancarboxaldehyde	C_6_H_6_O_2_	110.0	0.23	0.01	0.07	0.09	0.11	972.5	970
7	2,4-Dihydroxy-2,5-dimethyl-3(2*H*)-furan-3-one	C_6_H_8_O_4_	144.0	0.71	0.06	0.49		0.20	986.5	989
8	2-methyl-1,3-Cyclopentanedione	C_6_H_8_O_2_	112.0	0.51			0.29	0.09	1003.6	1003
9	Furaneol	C_6_H_8_O_3_	128.1	0.15	0.05	0.20	0.07	0.10	1071.6	1073
10	2-Furancarboxylic acid	C_5_H_4_O_3_	112.0			0.31	0.35		1091.0	1088
11	Phenylethyl alcohol	C_8_H_10_O	122.1		0.05				1120.8	1117
12	2,3-dihydro-3,5-dihydroxy-6-methyl-4*H*-pyran-4-one	C_6_H_8_O_4_	144.0	10.60	0.26	3.03	1.35	1.42	1153.9	1153
13	Benzoic acid	C_7_H_6_O_2_	122.1	0.72	0.29				1175.2	1178
14	Catechol	C_6_H_6_O_2_	110.1	0.02					1215.3	1219
15	2,3-dihydro-Benzofuran	C_8_H_8_O	120.1		0.30	0.38	3.63	3.31	1226.7	1226
16	5-Hydroxymethylfurfural	C_6_H_6_O_3_	126.0	1.48					1235.2	1234
17	Benzeneacetic acid	C_8_H_8_O_2_	136.1		0.23				1259.5	1257
18	Malic acid	C_4_H_6_O_5_	134.0		1.03		1.19	1.52	1266.8	MS ^(3)^
19	Indole	C_8_H_7_N	117.0		0.03				1301.6	1300
20	2-Methoxy-4-vinylphenol	C_9_H_10_O_2_	150.1		0.80	0.18	0.08	0.05	1319.9	1317
21	Eugenol	C_10_H_12_O_2_	164.1		0.30	0.20	0.38	0.37	1362.9	1362
22	1,2,3-Benzenetriol	C_6_H_6_O_3_	126.0		23.87	13.45	28.31	27.15	1398.5	1386
23	3-hydroxy-4-methoxy-Benzaldehyde	C_8_H_8_O_3_	152.0	0.64					1404.6	1401
24	2-methoxyphenyl-Ethanol	C_9_H_12_O_2_	152.1	0.31					1439.5	1421
25	2,4-di-tert-butylphenol	C_14_H_22_O	206.2	0.52					1512.1	1513
26	4-hydroxy-Benzoic acid	C_7_H_6_O_3_	138.0	0.56		0.22	0.59	0.58	1537.3	1538
27	Dodecanoic acid	C_12_H_24_O_2_	200.1				0.17		1557.9	1557
28	3-Methoxy-4-Hydroxybenzoic acid (Vanillic acid)	C_8_H_8_O_4_	168.0	1.38		0.27	0.30	0.54	1572.5	1570
29	4-hydroxy-3-methoxy-Benzoic acid, ethyl ester	C_10_H_12_O_4_	196.1				0.03		1590.1	1589
30	Tridecanoic acid	C_13_H_26_O_2_	214.2	17.76					1652.4	1662
31	Tetradecanoic acid	C_14_H_28_O_2_	228.2	0.51		0.36			1755.3	1761
32	6-Hydroxy-4,4,7a-trimethyl-5,6,7,7a-tetrahydrobenzofuran-2(4*H*)-one (Loliolide)	C_11_H_16_O_3_	196.1		0.21				1784.8	1784
33	4-Acetoxycinnamic acid	C_11_H_10_O_4_	206.1				0.74	0.22	1805.9	MS
34	Pentadecanoic acid	C_15_H_30_O_2_	242.2			0.20			1854.0	1857
35	2-methyl-Undecanoic acid, methyl ester	C_13_H_26_O_2_	214.2			0.06			1884.9	MS ^(3)^
36	Methyl gallate	C_8_H_8_O_5_	184.0				1.79	3.24	1914.6	MS ^(3)^
37	n-Hexadecanoic acid	C_16_H_32_O_2_	256.2	10.21	0.31	6.38	1.40	3.01	1960.3	1957
38	Ethyl gallate	C_9_H_10_O_5_	198.2	9.84	60.25	24.49	38.63	36.68	1978.0	MS ^(3)^
39	Hexadecanoic acid, ethyl ester (Ethyl palmitate)	C_18_H_36_O_2_	284.3	5.23					1985.0	1984
40	Heptadecanoic acid	C_17_H_34_O_2_	270.3			0.23			2054.3	2065
41	Phytol	C_20_H_40_O	296.3		0.18	1.80	0.25	0.52	2108.3	2112
42	(*Z*,*Z*)-9,12-Octadecadienoic acid	C_18_H_32_O_2_	280.2	3.07		2.80	0.71		2131.4	2130
43	(*Z*,*Z*,*Z*)-9,12,15-Octadecatrienoic acid	C_18_H_30_O_2_	278.2					1.38	2135.4	2143
44	9-Octadecenoic acid	C_18_H_34_O_2_	282.3	2.91		3.60			2139.7	2141
45	Linoleic acid ethyl ester	C_20_H_36_O_2_	308.3	3.38		4.26	1.06	2.17	2155.7	2155
46	9-Octadecenoic acid, ethyl ester	C_20_H_38_O_2_	310.3	3.22		2.35			2160.9	2171
47	(*Z*,*Z*,*Z*)-9,12,15-Octadecatrienoic acid, ethyl ester	C_20_H_34_O_2_	306.3	0.59		1.64	0.40	0.77	2163.2	2169
48	Octadecanoic acid	C_18_H_36_O_2_	284.3	0.68	0.19	0.30	0.55	0.95	2174.5	2172
49	Octadecanoic acid, ethyl ester	C_20_H_40_O_2_	312.3	0.74	0.60				2183.1	2188
50	(*Z*)-9-Octadecenamide	C_18_H_35_NO	281.3	9.00	1.65	3.52	5.57	9.33	2364.0	2375
51	Eicosanoic acid, ethyl ester	C_22_H_44_O_2_	340.3	1.30		0.57	0.20	0.41	2382.2	2394
52	Hexadecanoic acid, 2-hydroxy-1-(hydroxymethyl)ethyl ester	C_19_H_38_O_4_	330.3	3.25		0.73		0.49	2501.6	2498
53	Docosanoic acid, ethyl ester	C_24_H_48_O_2_	368.6			0.51			2586.3	2594

^(1)^ KI_exp_: Kovats indices calculated from retention time data on a HP-5ms capillary column. ^(2)^ KI_lit_: Kovats indices from literature (NIST). ^(3)^ The Kovats index was not reported on a HP-5ms capillary column, the identification was made by comparison with M.S. mass spectra.

**Table 5:** M.I.C. of FDE-Flower and FDE-Leaf partitioned extracts.

	Solvent			
Strain	Chl	EtOAc	MeOH	FDE
*S. aureus*	250 µg/mL	31.2 µg/mL	>1000 µg/mL	Leaf
	1000 µg/mL	31.2 µg/mL	1000 µg/mL	Flower
*S. enterica*	1000 µg/mL	62.5 µg/mL	>1000 µg/mL	Leaf
	250 µg/mL	62.5 µg/mL	1000 µg/mL	Flower
*E. faecalis*	1000 µg/mL	62.5 µg/mL	>1000 µg/mL	Leaf
	500 µg/mL	31.2 µg/mL	1000 µg/mL	Flower
*S. marcescens*	>1000 µg/mL	500 µg/mL	>1000 µg/mL	Leaf
	1000 µg/mL	500 µg/mL	>1000 µg/mL	Flower

**Table 6:** Volatile and semi-volatile compounds identified in leaf and flower ethyl acetate fractions of *Pelargonium peltatum* by GC-MS.

#	Name	MF	MW	% Area		KI_exp_^(1)^	KI_lit_^(2)^
				Leaf	Flower		
1	Furfural	C_5_H_4_O_2_	96.0		0.11	855.1	852
2	3-methyl-2,5-furandione	C_5_H_4_O_3_	112.0	0.02	0.03	952.0	949
3	Acetophenone	C_8_H_8_O	120.0	0.04		1070.4	1066
4	2-methoxy-Phenol	C_7_H_8_O_2_	124.1		0.06	1091.5	1090
5	Nonanal	C_9_H_18_O	142.1	0.07		1114.7	1112
6	Catechol	C_6_H_6_O_2_	110.0		0.13	1200.2	1208
7	2,3-dihydro-Benzofuran	C_8_H_8_O	120.1	0.04	1.59	1222.4	1226
8	5-Hydroxymethylfurfural	C_6_H_6_O_3_	126.0		0.10	1256.4	1261
9	2-Methoxy-4-vinylphenol	C_9_H_10_O_2_	150.1	0.02	0.02	1314.5	1317
10	Eugenol	C_10_H_12_O_2_	164.1		0.05	1358.1	1362
11	1,2,3-Benzenetriol	C_6_H_6_O_3_	126.0	77.38	71.24	1387.4	1386
12	2,4-di-tert-butylphenol	C_14_H_22_O	206.2	0.09		1505.9	1513
13	Ethyl gallate	C_9_H_10_O_5_	198.1		13.10	1951.3	MS
14	Octadecanoic acid	C_18_H_36_O_2_	284.3	1.44	0.54	2167.2	2172
15	(Z)-9-Octadecenamide	C_18_H_35_NO	281.3	13.63	6.75	2364.0	2375

^(1)^ KI_exp_: Kovats indices calculated from retention time data on a HP-5ms capillary column. ^(2)^ KI_lit_: Kovats indices from literature (NIST). The Kovats index was not reported on a HP-5ms capillary column, the identification was made by comparison with M.S. (mass spectra).

## Data Availability

Not applicable.

## Supplementary Materials

The following supporting information can be downloaded at: https://www.mdpi.com/article/10.3390/molecules27113436/s1, Figure S1: Thin layer chromatography of different freeze-dried extracts from *P. peltatum*; Figure S2: Evaluation of the antimicrobial activity of *P. peltatum* FDE against; Figure S3: Evaluation of the antimicrobial activity of FDE-flowers and FDE-Leaf fractions; Table S1: Solubility of *P. peltatum* freeze-dried extracts; Table S2: Inhibition zone growth (mm) of FDEs against different strains.

## References

[1] Kliebenstein D.J., Osbourn A. (2012). Making New Molecules—Evolution of Pathways for Novel Metabolites in Plants. Curr. Opin. Plant Biol..

[2] Isah T. (2019). Stress and Defense Responses in Plant Secondary Metabolites Production. Biol. Res..

[3] Rai A., Saito K., Yamazaki M. (2017). Integrated Omics Analysis of Specialized Metabolism in Medicinal Plants. Plant J..

[4] Erb M., Kliebenstein D.J. (2020). Plant Secondary Metabolites as Defenses, Regulators, and Primary Metabolites: The Blurred Functional Trichotomy. Plant Physiol..

[5] Platzer M., Kiese S., Herfellner T., Schweiggert-Weisz U., Miesbauer O., Eisner P. (2021). Common Trends and Differences in Antioxidant Activity Analysis of Phenolic Substances Using Single Electron Transfer Based Assays. Molecules.

[6] Durazzo A., Lucarini M., Souto E.B., Cicala C., Caiazzo E., Izzo A.A., Novellino E., Santini A. (2019). Polyphenols: A Concise Overview on the Chemistry, Occurrence, and Human Health. Phyther. Res..

[7] Gutiérrez-del-Río I., Fernández J., Lombó F. (2018). Plant Nutraceuticals as Antimicrobial Agents in Food Preservation: Terpenoids, Polyphenols and Thiols. Int. J. Antimicrob. Agents.

[8] Mierziak J., Kostyn K., Kulma A. (2014). Flavonoids as Important Molecules of Plant Interactions with the Environment. Molecules.

[9] Pizzi A. (2019). Tannins: Prospectives and Actual Industrial Applications. Biomolecules.

[10] Flieger J., Flieger W., Baj J., Maciejewski R. (2021). Antioxidants: Classification, Natural Sources, Activity/Capacity Measurements, and Usefulness for the Synthesis of Nanoparticles. Materials.

[11] Du L., Liu W. (2012). Occurrence, Fate, and Ecotoxicity of Antibiotics in Agro-Ecosystems. A Review. Agron. Sustain. Dev..

[12] Ara I., Shinwari M.M.A., Rashed S.A., Bakir M.A. (2013). Evaluation of Antimicrobial Properties of Two Different Extracts of Juglans Regia Tree Bark and Search for Their Compounds Using Gas Chromatohraphy-Mass Spectrum. Int. J. Biol..

[13] Maree J., Kamatou G., Gibbons S., Viljoen A., Van Vuuren S. (2014). The Application of GC-MS Combined with Chemometrics for the Identification of Antimicrobial Compounds from Selected Commercial Essential Oils. Chemom. Intell. Lab. Syst..

[14] Valle D.L., Puzon J.J.M., Cabrera E.C., Rivera W.L. (2016). Thin Layer Chromatography-Bioautography and Gas Chromatography-Mass Spectrometry of Antimicrobial Leaf Extracts from Philippine *Piper Betle* L. against Multidrug-Resistant Bacteria. Evidence-Based Complement. Altern. Med..

[15] Wagner K., Roth C., Willför S., Musso M., Petutschnigg A., Oostingh G.J., Sclmabel T. (2019). Identification of Antimicrobial Compounds in Different Hydrophilic Larch Bark Extracts. BioResources.

[16] Safdar M., Naqvi S.A., Anjum F., Pasha I., Shahid M., Waliullah, Jaskani M.J., Khan I.A., Aadil R.M. (2021). Microbial Biofilm Inhibition, Antioxidants, and Chemical Fingerprints of Afghani Pomegranate Peel Extract Documented by Gas Chromatography–Mass Spectrometry and Fourier Transformation Infrared. J. Food Process. Preserv..

[17] Park Y.J., Baskar T.B., Yeo S.K., Arasu M.V., Al-Dhabi N.A., Lim S.S., Park S.U. (2016). Composition of Volatile Compounds and in Vitro Antimicrobial Activity of Nine *Mentha* Spp.. Springerplus.

[18] Taherpour§ A., Maroofi H., Kheradmand K. (2007). Chemical Composition of the Essential Oil of Pelargonium Quercetorum Agnew. of Iran. Nat. Prod. Res..

[19] Al-Nemari R., Al-Senaidy A., Semlali A., Ismael M., Badjah-Hadj-Ahmed A.Y., Ben Bacha A. (2020). GC-MS Profiling and Assessment of Antioxidant, Antibacterial, and Anticancer Properties of Extracts of *Annona Squamosa* L. Leaves. BMC Complement. Med. Ther..

[20] Viet T.D., Xuan T.D., Van T.M., Andriana Y., Rayee R., Tran H.-D. (2019). Comprehensive Fractionation of Antioxidants and GC-MS and ESI-MS Fingerprints of Celastrus Hindsii Leaves. Medicines.

[21] Ben ElHadj Ali I., Tajini F., Boulila A., Jebri M.A., Boussaid M., Messaoud C., Sebaï H. (2020). Bioactive Compounds from Tunisian *Pelargonium Graveolens* (L’Hér.) Essential Oils and Extracts: α-Amylase and Acethylcholinesterase Inhibitory and Antioxidant, Antibacterial and Phytotoxic Activities. Ind. Crops Prod..

[22] Guerrini A., Rossi D., Paganetto G., Tognolini M., Muzzoli M., Romagnoli C., Antognoni F., Vertuani S., Medici A., Bruni A. (2011). Chemical Characterization (GC/MS and NMR Fingerprinting) and Bioactivities of South-African Pelargonium Capitatum (L.) L’ Her. (Geraniaceae) Essential Oil. Chem. Biodivers..

[23] Maree J.E., Viljoen A.M. (2012). Phytochemical Distinction between Pelargonium Sidoides and Pelargonium Reniforme—A Quality Control Perspective. S. Afr. J. Bot..

[24] Theisen L.L., Gohrbandt S., Muller C.P., Luetteke N. (2012). EPs^®^ 7630 (Umckaloabo^®^), an Extract from Pelargonium Sidoides Roots, Exerts Anti-Influenza Virus Activity in Vitro and in Vivo. Int. J. Infect. Dis..

[25] Kayser O., Kolodziej H. (1997). Antibacterial Activity of Extracts and Constituents of Pelargonium Sidoides and Pelargonium Reniforme. Planta Med..

[26] Lewu F.B., Grierson D.S., Afolayan A.J. (2006). Extracts from Pelargonium Sidoides Inhibit the Growth of Bacteria and Fungi. Pharm. Biol..

[27] Papies J., Emanuel J., Heinemann N., Kulić Ž., Schroeder S., Tenner B., Lehner M.D., Seifert G., Müller M.A. (2021). Antiviral and Immunomodulatory Effects of *Pelargonium Sidoides DC*. Root Extract EPs^®^ 7630 in SARS-CoV-2-Infected Human Lung Cells. Front. Pharmacol..

[28] Pereira A., Bester M., Soundy P., Apostolides Z. (2016). Anti-Proliferative Properties of Commercial Pelargonium Sidoides Tincture, with Cell-Cycle G0/G1 Arrest and Apoptosis in Jurkat Leukaemia Cells. Pharm. Biol..

[29] Terlizzi M., Colarusso C., Di Maio U., Bagnulo A., Pinto A., Sorrentino R. (2020). Antioxidant and Antimicrobial Properties of Pelargonium Sidoides DC and Lactoferrin Combination. Biosci. Rep..

[30] Kolodziej H., Kayser O., Radtke O.A., Kiderlen A.F., Koch E. (2003). Pharmacological Profile of Extracts of Pelargonium Sidoides and Their Constituents. Phytomedicine.

[31] Jeiter J., Hilger H.H., Smets E.F., Weigend M. (2017). The Relationship between Nectaries and Floral Architecture: A Case Study in Geraniaceae and Hypseocharitaceae. Ann. Bot..

[32] Bakker F.T., Culham A., Hettiarachi P., Touloumenidou T., Gibby M. (2004). Phylogeny of Pelargonium (Geraniaceae) Based on DNA Sequences from Three Genomes. Taxon.

[33] Ullah A., Munir S., Badshah S.L., Khan N., Ghani L., Poulson B.G., Emwas A.H., Jaremko M. (2020). Important Flavonoids and Their Role as a Therapeutic Agent. Molecules.

[34] Ghannadi A., Bagherinejad M.R., Abedi D., Jalali M., Absalan B., Sadeghi N. (2012). Antibacterial Activity and Composition of Essential Oils from *Pelargonium Graveolens* L’Her and *Vitex Agnus-Castus* L.. Iran. J. Microbiol..

[35] Williams V.L., Victor J.E., Crouch N.R. (2013). Red Listed Medicinal Plants of South Africa: Status, Trends, and Assessment Challenges. South African J. Bot..

[36] Ibrahim M.M., El Ghani S.A., El-Moez S.I.A. (2018). Phytochemical Analysis and Antimicrobial Activities of Different Callus Extracts of Pelargonium Sidoides DC. against Food Borne Pathogenic Bacteria. J. Appl. Pharm. Sci..

[37] Ennaifer M., Bouzaiene T., Chouaibi M., Hamdi M. (2018). *Pelargonium Graveolens* Aqueous Decoction: A New Water-Soluble Polysaccharide and Antioxidant-Rich Extract. Biomed Res. Int..

[38] Karatoprak G.Ş., Göger F., Yerer M.B., Koşar M. (2017). Chemical Composition and Biological Investigation of Pelargonium Endlicherianum Root Extracts. Pharm. Biol..

[39] Leri M., Scuto M., Ontario M.L., Calabrese V., Calabrese E.J., Bucciantini M., Stefani M. (2020). Healthy Effects of Plant Polyphenols: Molecular Mechanisms. Int. J. Mol. Sci..

[40] Asuzu P.C., Besong S.A., Aryee A.N. (2019). Polyphenols and Other Phytochemicals in Cancer Prevention and Management. FASEB J..

[41] Proestos C., Varzakas T. (2017). Aromatic Plants: Antioxidant Capacity and Polyphenol Characterisation. Foods.

[42] Abdelli M., Moghrani H., Aboun A., Maachi R. (2016). Algerian Mentha Pulegium L. Leaves Essential Oil: Chemical Composition, Antimicrobial, Insecticidal and Antioxidant Activities. Ind. Crops Prod..

[43] Xu D.P., Li Y., Meng X., Zhou T., Zhou Y., Zheng J., Zhang J.J., Li H. (2017). Bin Natural Antioxidants in Foods and Medicinal Plants: Extraction, Assessment and Resources. Int. J. Mol. Sci..

[44] Yong-Bing X., Gui-Lin C., Ming-Quan G. (2019). Antioxidant and Anti-Inflammatory Activities of the Crude Extracts of Moringa Oleifera from Kenya and Their Correlations with Flavonoids. Antioxidants.

[45] Hsu F.L., Huang W.J., Wu T.H., Lee M.H., Chen L.C., Lu H.J., Hou W.C., Lin M.H. (2012). Evaluation of Antioxidant and Free Radical Scavenging Capacities of Polyphenolics from Pods of Caesalpinia Pulcherrima. Int. J. Mol. Sci..

[46] Okoh S.O., Asekun O.T., Familoni O.B., Afolayan A.J. (2014). Antioxidant and Free Radical Scavenging Capacity of Seed and Shell Essential Oils Extracted from *Abrus Precatorius* (L). Antioxidants.

[47] Cynthia, Florence I., Hery S., Akhmad D. (2018). Antibacterial and Antioxidant Activities of Pyrogallol and Synthetic Pyrogallol Dimer. Res. J. Chem. Environ..

[48] Kocaçalişkan I., Talan I., Terzi I. (2006). Antimicrobial Activity of Catechol and Pyrogallol as Allelochemicals. Z. Naturforsch. Sect. C J. Biosci..

[49] Shao J., Wu Z., Yu G., Peng X., Li R. (2009). Allelopathic Mechanism of Pyrogallol to Microcystis Aeruginosa PCC7806 (Cyanobacteria): From Views of Gene Expression and Antioxidant System. Chemosphere.

[50] Lima V.N., Oliveira-Tintino C.D.M., Santos E.S., Morais L.P., Tintino S.R., Freitas T.S., Geraldo Y.S., Pereira R.L.S., Cruz R.P., Menezes I.R.A. (2016). Antimicrobial and Enhancement of the Antibiotic Activity by Phenolic Compounds: Gallic Acid, Caffeic Acid and Pyrogallol. Microb. Pathog..

[51] Shin M., Park E., Lee H. (2019). Plant-Inspired Pyrogallol-Containing Functional Materials. Adv. Funct. Mater..

[52] Johnstone D.B., Little J.E. (1953). Bacteriostatic, Bactericidal, and Drug Resistance Studies of Ethyl Gallate on Mycobacterium Tuberculosis. J. Bacteriol..

[53] Mink S.N., Jacobs H., Gotes J., Kasian K., Cheng Z.Q. (2011). Ethyl Gallate, a Scavenger of Hydrogen Peroxide That Inhibits Lysozyme-Induced Hydrogen Peroxide Signaling in Vitro, Reverses Hypotension in Canine Septic Shock. J. Appl. Physiol..

[54] Oladimeji O.H., Igboasoiyi A. (2014). Isolation, Characterization and Antimicrobial Analysis of Ethyl Gallate and Pyrogallol from Acalypha Wilkesiana Var. Lace-Acalypha (Muell &Arg.). Afr. J. Pharmacol. Ther..

[55] Ooshiro A., Hiradate S., Kawano S., Takushi T., Fujii Y., Natsume M., Abe H. (2009). Identification and Activity of Ethyl Gallate as an Antimicrobial Compound Produced by Geranium Carolinianum. Weed Biol. Manag..

[56] Hall G., Le T.T.T., Stanford J.B., Sugden J.K. (1996). Hydroxyl Radical Scavenging by Ethyl Gallate and Related Compounds: A Method for Rapid Evaluation. Pharm. Acta Helv..

[57] Hausen B.M., Beyer W. (1992). The Sensitizing Capacity of the Antioxidants Propyl, Octyl, and Dodecyl Gallate and Some Related Gallic Acid Esters. Contact Dermatitis.

[58] Osman A., Hamed A., Mohamed S., Ayoub H. (2015). Chemical Composition and Antimicrobial Activity of Sudanese Lupinus Termis L. root extracts. Pharma Innov. J..

[59] Driscoll W.J., Chaturvedi S., Mueller G.P. (2007). Oleamide Synthesizing Activity from Rat Kidney: Identification as Cytochrome C. J. Biol. Chem..

[60] Nischitha R., Shivanna M.B. (2021). Antimicrobial Activity and Metabolite Profiling of Endophytic Fungi in *Digitaria Bicornis* (Lam) Roem. and Schult. and *Paspalidium Flavidum* (Retz.) A. Camus. 3 Biotech.

[61] Lourenço S.C., Moldão-Martins M., Alves V.D. (2019). Antioxidants of Natural Plant Origins: From Sources to Food Industry Applications. Molecules.

[62] Brand-Williams W., Cuvelier M.E., Berset C. (1995). Use of a Free Radical Method to Evaluate Antioxidant Activity. LWT Food Sci. Technol..

[63] Li H.-B., Wong C.C., Cheng K.W., Chen F. (2008). Antioxidant Properties in Vitro and Total Phenolic Contents in Methanol Extracts from Medicinal Plants. LWT Food Sci. Technol..

[64] Benzie I.F.F., Strain J.J. (1996). The Ferric Reducing Ability of Plasma (FRAP) as a Measure of “Antioxidant Power”: The FRAP Assay. Anal. Biochem..

[65] Singleton V.L., Orthofer R., Lamuela-Raventós R.M. (1999). [14] Analysis of Total Phenols and Other Oxidation Substrates and Antioxidants by Means of Folin-Ciocalteu Reagent. Methods in Enzymology.

[66] Broadhurst R.B., Jones W.T. (1978). Analysis of Condensed Tannins Using Acidified Vanillin. J. Sci. Food Agric..

[67] Zhishen J., Mengcheng T., Jianming W. (1999). The Determination of Flavonoid Contents in Mulberry and Their Scavenging Effects on Superoxide Radicals. Food Chem..

[68] Lucero M., Estell R., Tellez M., Fredrickson E. (2009). A Retention Index Calculator Simplifies Identification of Plant Volatile Organic Compounds. Phytochem. Anal..

[69] Reza A.S.M.A., Haque M.A., Sarker J., Nasrin M.S., Rahman M.M., Tareq A.M., Khan Z., Rashid M., Sadik M.G., Tsukahara T. (2021). Antiproliferative and Antioxidant Potentials of Bioactive Edible Vegetable Fraction of *Achyranthes Ferruginea* Roxb. in Cancer Cell Line. Food Sci. Nutr..

