# Supplementary material for: GC/MS Analysis, Antioxidant Activity, and Antimicrobial Effect of Pelargonium peltatum (Geraniaceae)
Source: Molecules. 2022 May 26;27(11):3436. doi: 10.3390/molecules27113436 (PMC9181846; doi:10.3390/molecules27113436)
Supplement: Supplementary file 1 [file molecules-27-03436-s001.zip › molecules-1646416-supplementary.pdf]

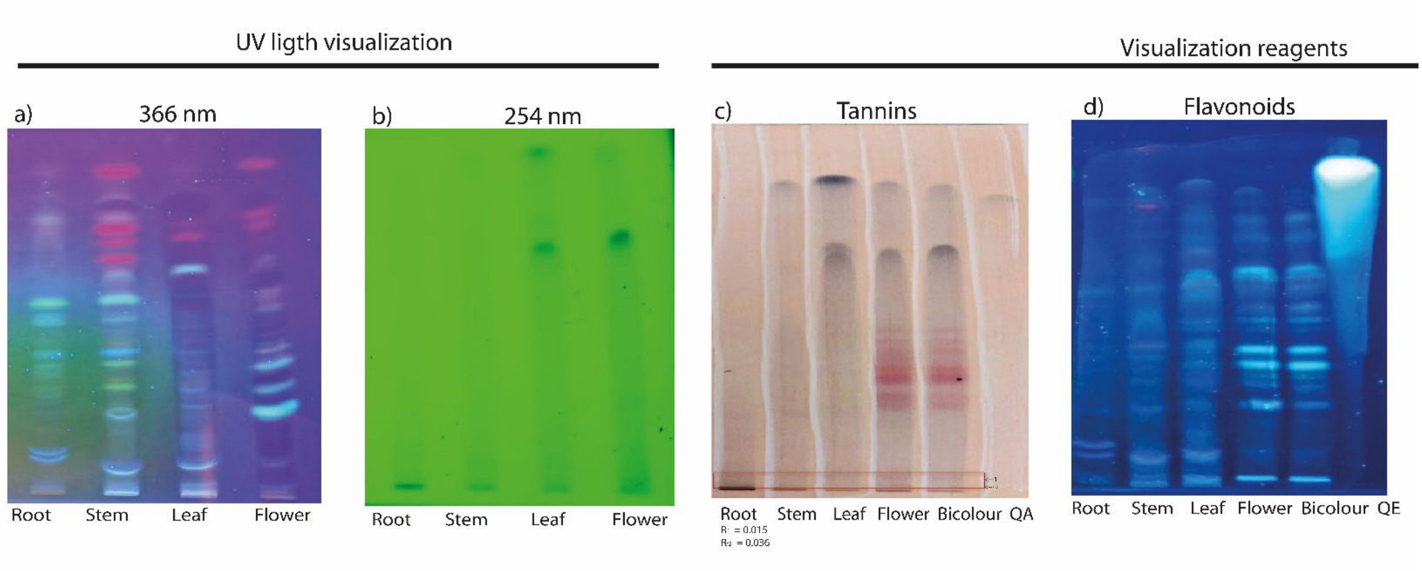

Figure S1. Thin layer chromatography of Different freeze-dried extracts from *P. peltatum*. A) TLC visualize by UV light at 366 nm, B) TLC visualize by UV light at 254 nm, C) Tannins revealed by 1%, Ferric chloride D) Flavonoids revealed by 1 % of Aluminum chloride, and E) Reducing Sugars revealed by trichloroacetic acid.

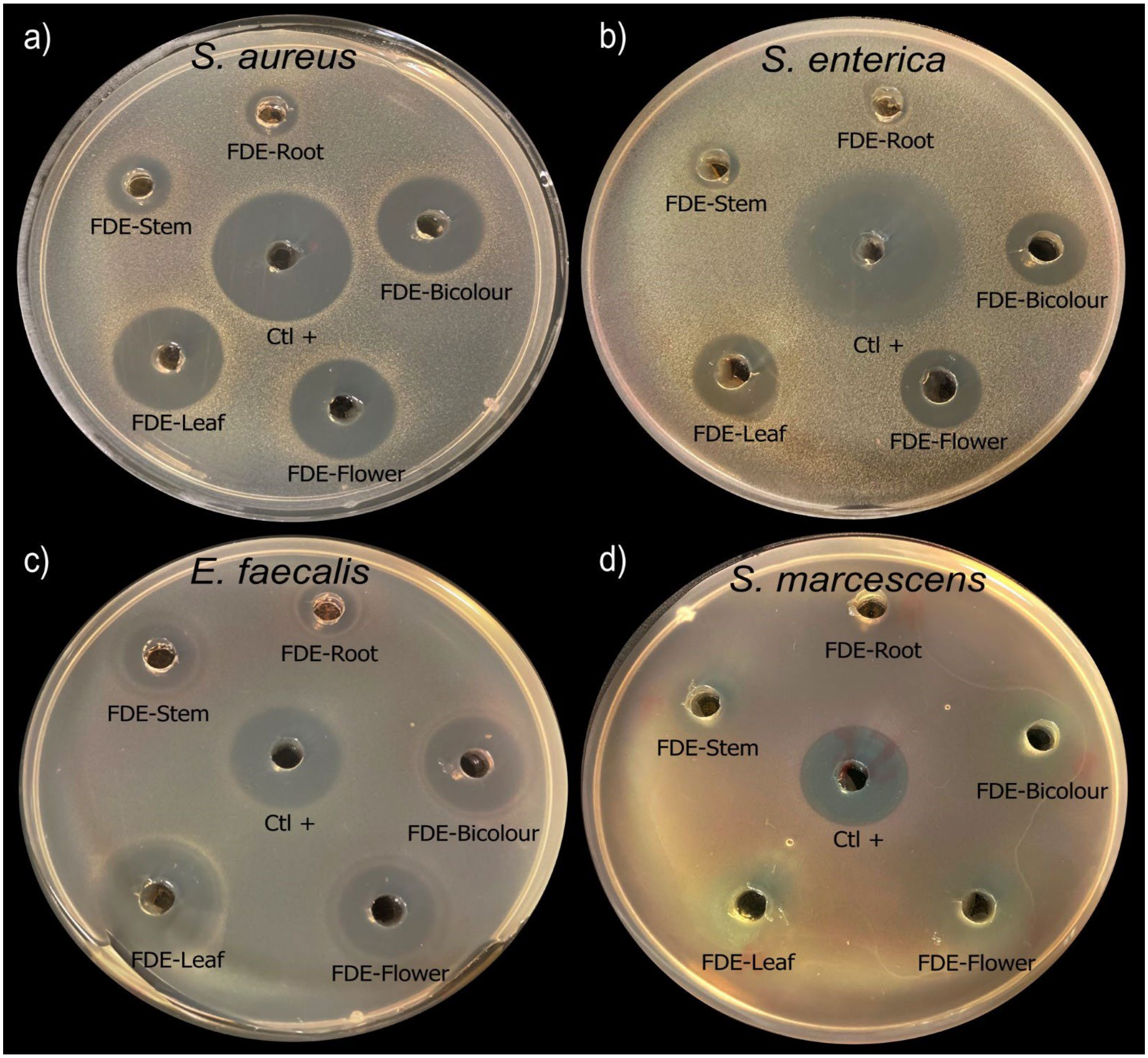

Figure S2. Evaluation of the antimicrobial activity of *P. peltatum* FDE against : a) *S. aureus*, b) *S. enterica*, c) *E. faecalis* and d) *S. marcescens*. Ctl+: Kanamycin positive control.

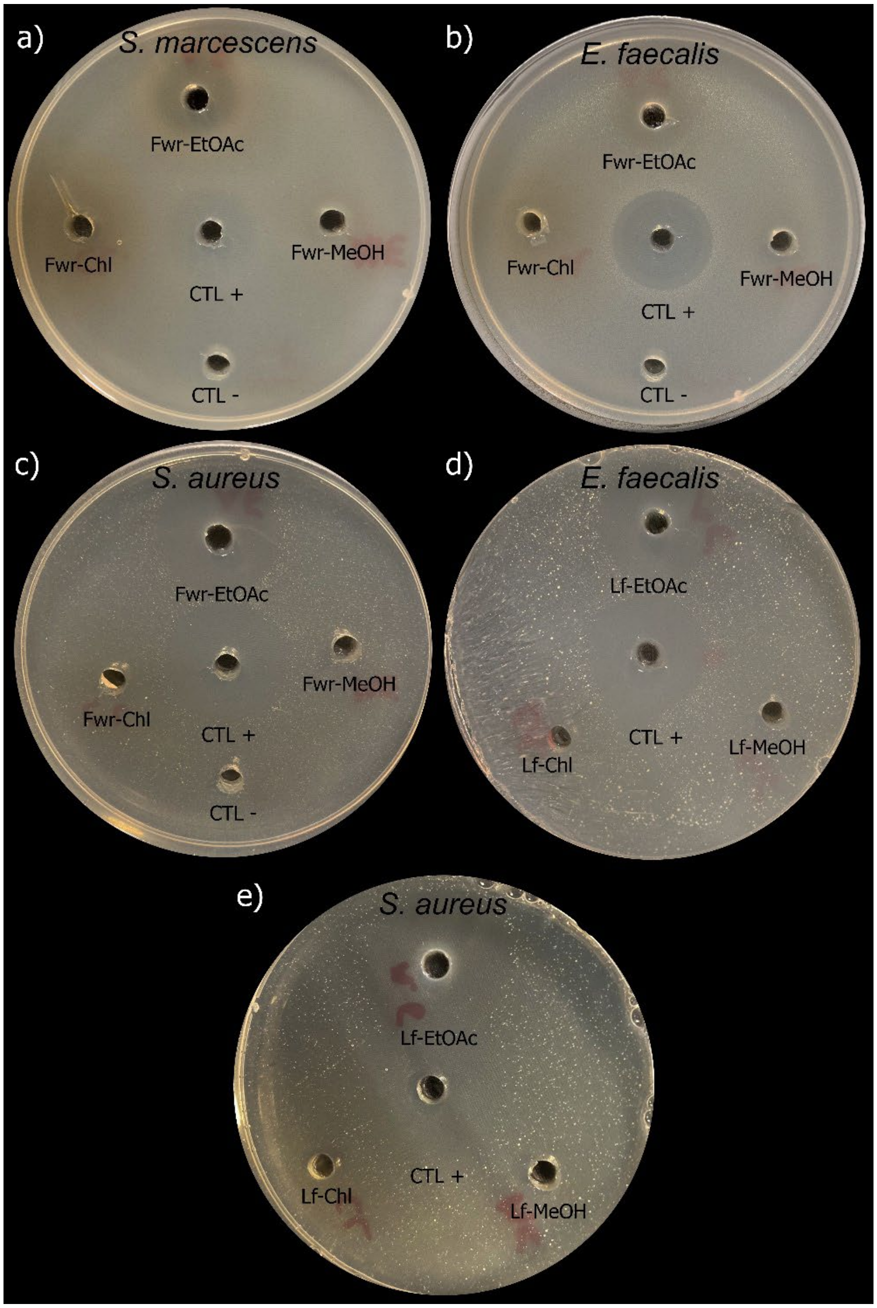

Figure S3. Evaluation of the antimicrobial activity of FDE-flowers and FDE-Leaf fractions against a) *S. marcescens,* b) *E. faecalis, c) S. aureus*, d) *E. faecalis and e) S. aureus.* Ctl+: Kanamycin positive control. Clt -: LB with 2% of DMSO. Fractions were resuspended in sterile LB with 2% of DMSO.

Table S1. Solubility of *P. peltatum* freeze-dried extracts.

Polarity	Solvent	FDE-Root	FDE-Stem	FDE-Leaf	FDE-Flower
	Water	++	++	+	++
Polar	Ethanol 96%	-	+	+	+
	Ethanol 70%	++	++	++	+
	Methanol	++	++	++	+
	Chloroform	+	+	+	-
	Dichloromethane	+	+	-	-
	Ethyl acetate	-	+	-	-
Non polar	Hexane	-	-	-	-
	Diethyl ether	-	+	+	-

(-) Non soluble; (+) Soluble; (++) Very soluble

Table S2. Inhibition zone growth (mm) of FDEs against different strains

Strain	FDE-Root (mm)	FDE-Stem (mm)	FDE-Leaf (mm)	FDE-Flower (mm)	FDE-Bicolor (mm)	Control + (mm)
S. aureus	9.89 ± 0.30 **abc**	12.15 ± 0.67 **def**	22.15 ± 0.71 **g**	20.33 ± 0.75	19.67 ± 0.53	26.19 ± 0.72
S. enterica	7.57 ± 1.48 **ac**	8.85 ± 0.79 **d**	12.29 ± 1.07 **h**	12.36 ± 1.04	10.76 ± 1.44	26.57 ± 0.92
E. faecalis	11.3 ± 0.27 **abc**	14.33 ± 0.79 **d**	22.41 ± 1.31 **gh**	18.41 ± 0.96	18.78 ± 1.43	20.93 ± 1.28
S. marcenses.	9.60 ± 0.47 **abc**	11.01 ± 0.34 **de**	15.83 ± 0.50 **g**	16.01 ± 0.44 **i**	16.40 ± 0.39	19.86 ± 0.36

Plus/minus values are means ± S.D.

† p-values for differences among the five extracts were calculated by analysis of variance. When the difference among the regions was significant ($p < 0.05$), all pairwise comparisons tested for significance with the Tukey's Honest Significant Difference test (TukeyHSD) procedure.

‡ The value for the variable in FDE-Root is significantly different from the value in FDE-Stem ($p < 0.001$).

a The value for the variable in FDE-Root is significantly different from the value in FDE-Leaf ($p < 0.001$).

b The value for the variable in FDE-Root is significantly different from the value in FDE-Flower ($p < 0.001$).

c The value for the variable in FDE-Root is significantly different from the value in FDE-Bicolor ($p < 0.001$)

d The value for the variable in FDE-Stem is significantly different from the value in FDE-Leaf ($p < 0.001$).

e The value for the variable in FDE-Stem is significantly different from the value in FDE-Flower ($p < 0.001$)

f The value for the variable in FDE-Stem is significantly different from the value in FDE-Bicolor ($p < 0.001$).

h The value for the variable in FDE-Leaf is significantly different from the value in FDE-Flower ($p < 0.05$).

g The value for the variable in FDE-Leaf is significantly different from the value in FDE-Bicolor ($p < 0.05$).

i The value for the variable inFDE-Flower is significantly different from the value in FDE-Bicolor ($p < 0.05$).

FDE, Freeze-Dried Extract; mm, milimeters
